# What Is Old Is New Again—Use of Augmented Reality in Craniometric Point Identification and Neuroanatomy Education

**DOI:** 10.1177/29941520251377066

**Published:** 2025-09-03

**Authors:** Joshua Olexa, Nick Caffes, Maureen Rakovec, Jordan R. Saadon, Ashish Sharma, Nicole Wenger, Annie Trang, Charles Sansur

**Affiliations:** Department of Neurosurgery, University of Maryland Medical Center, Baltimore, Maryland, USA.

**Keywords:** AR, craniometrics, neuroanatomy, headset, HoloLens, education

## Abstract

Traditional methods of teaching neurosurgery have often been inadequate in capturing the interest of students and maintaining their attention throughout the learning process. Augmented reality (AR) technology has been proposed as a solution to this problem by providing an interactive and immersive experience for students. In this article, we use an AR application on the Microsoft HoloLens 2 to educate students about neuroanatomy, promote greater interest and engagement with the field of neurosurgery, and facilitate communication between students and neurosurgeons. We present an exercise in which the AR application was used to educate high school students on skull-based craniometric landmarks commonly used by neurosurgeons. Students were asked to locate points on the skull with and without the assistance of AR and were surveyed about their experience using AR. The results suggest greater student engagement and a positive learning experience. Students were able to target craniometric points on the skull with greater accuracy using AR than without. Overall, students felt that incorporating AR into their education improved their ability to learn neuroanatomy and increased their interest in the field of neurosurgery. This study provides a pilot feasibility study with findings that suggest that AR has the potential to enhance the way neurosurgery and neuroanatomy are introduced to early learners through increased interaction and engagement.

## Introduction

Augmented reality (AR) has emerged as an innovative technology that is transforming education by creating immersive and interactive learning experiences. AR has been used in various fields, such as entertainment, gaming, and advertising, but its potential in education is enormous.^1,2^ AR can be an effective tool for increasing student interest in complex and challenging subjects such as anatomy, with even further educational power in understanding complex neuroanatomical structures of the brain and spine.^2–4^

Neurosurgery is a fascinating field that deals with the diagnosis and treatment of disorders relevant to neuroanatomical structures of the nervous system. However, it can be challenging to engage students in such a difficult subject matter, and traditional teaching methods may not be enough to capture their attention. By incorporating AR technology into the classroom, teachers can create interactive and visually stimulating learning environments that can increase student interest and long-term retention of knowledge in challenging topics such as neurosurgery and neuroanatomy. AR technology can provide students with a more realistic and hands-on approach to learning about the brain and nervous system. By using AR technology, students can visualize the inner workings of the brain, see how different parts of the brain interact with each other, and explore the various surgical techniques used in neurosurgery.^5,6^ For example, AR can be used to create 3D models of the brain and nervous system, allowing students to see the various parts of the brain and how they relate to each other. This can help students better understand the complex interplay between different parts of the brain and how they contribute to overall brain function.^7^

We developed an AR tool and employed this tool during an anatomy teaching session to educate high school students and increase their engagement within the field of neurosurgery. An exercise was developed whereby students were asked to identify key skull landmarks with and without the assistance of AR. Specifically, we evaluated the utility of AR for educating students about key craniometric landmarks on the skull.^8,9^ Students learned about key skull-based landmarks using traditional phantom models, cadaveric specimens, as well as AR. Our aim was to demonstrate increased understanding and visualization of neuroanatomical structures. In addition, we aimed to evaluate the impact that AR has as a learning tool to facilitate greater interest and engagement of students in the field of neurosurgery.

## Methods

### Technology platform

**FIG. 1.**
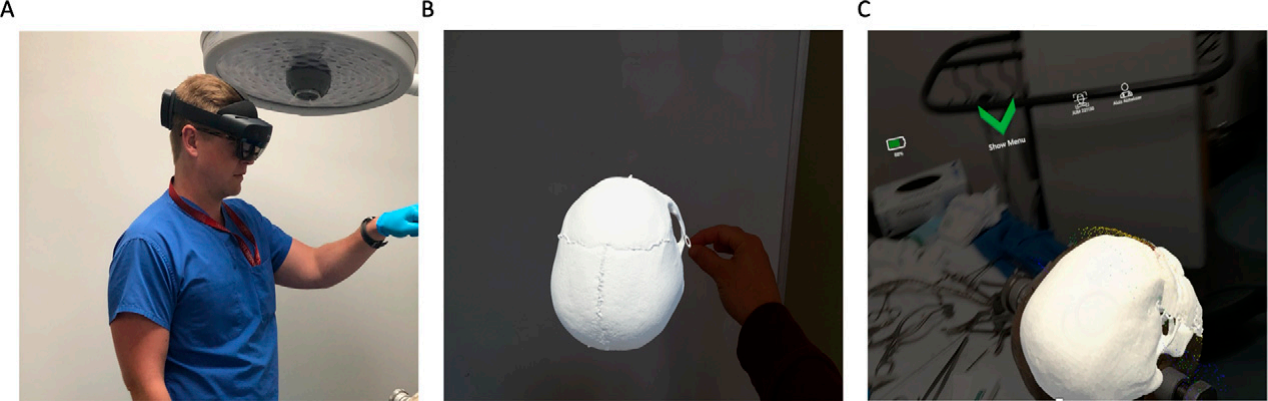
An AR application was developed on the Microsoft HoloLens 2 for visualizing the 3D reconstruction of a cadaver skull. Representative images of **(A)** user wearing the headset, **(B)** 3D model of cadaveric skull, and **(C)** 3D model of cadaveric skull overlaid onto cadaver head. AR, augmented reality.

A custom AR software application was used to display 3D models of cranial anatomy (Hoth Intelligence, Inc.). The software was developed for the Microsoft HoloLens 2 AR headset. Specifically, the software was used to display and superimpose a virtual 3D model of a cadaver skull onto a cadaveric head to allow participants to visualize the internal neuroanatomy (Fig. 1). To generate the 3D model, the cadaveric head underwent computed tomography (CT) scanning, and the resulting CT scan was converted into a 3D model (STL) file using proprietary 3D modeling software. For the purposes of the neuroanatomy lesson, specific craniometric points (called lambda and bregma) as well as the skull suture lines (coronal sutures and sagittal sutures) were outlined on the 3D reconstruction of the skull and overlaid on the cadaver head using the AR headset.

### Study outline

In order to educate students about craniometric points, a study was developed in which students were asked to mark the location of bregma and lambda on a cadaver head both with and without the assistance of AR. Prior to the start of the exercise, participants received education on the classical skull craniometric points lambda and bregma. Participants were first taught about these craniometric points by a neurosurgeon using a generic plastic skull to explain the location and importance of these structures, and how they are used practically by neurosurgeons for planning given neuroanatomic relevance. After didactic instruction, the participants were asked to mark the location of bregma and lambda on the cadaveric head based on classical teaching methods. (Note: The skull was not exposed; therefore, participants had little guidance to locate these structures other than traditional methods based on palpation and measurement.) Next, while wearing the AR headset, the participants visualized the cadaver head with the 3D skull model overlaid and were asked again to mark the location of lambda and bregma. The true locations of these points were determined prior to the start of the study based on accurate measurements made from evaluation of CT imaging of the cadaveric head.

### Participants

**FIG. 2.**
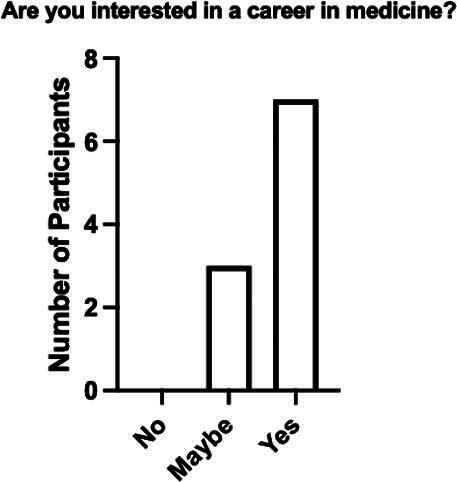
Survey results of participants’ interest in a career in medicine.

**FIG. 3.**
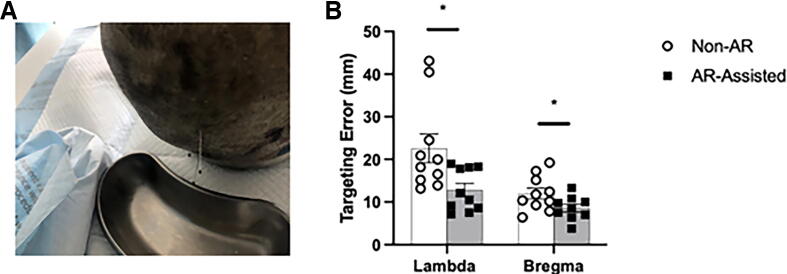
**(A)** Representative image of participant markings on craniometric points. **(B)** Accuracy of targeting craniometric points lambda and bregma, with and without AR assistance. Error bars represent standard error of the mean. *p < 0.05.

A total of 10 participants were enrolled in the study. Participants were high school students in their senior year actively involved in a neuroscience class with varying degrees of interest in a career in medicine (Fig. 2). All participants were in both the non-AR-assisted and AR-assisted groups. None of the participants had experience with identifying human craniometric points prior to the study (Fig. 3).

### Accuracy of craniometric point targeting

Participants’ ability to locate these skull landmarks was determined based on the distance between the participants’ marked point and the known location of the landmark (determined by neurosurgeon using precise measurements from CT scan). Data are reported as the targeting error in millimeter.

### Qualitative survey

At the conclusion of the study, all participants completed a survey about the perceived value of AR for teaching neuroanatomy. Additionally, the survey evaluated the impact of AR on students’ overall experience and interest in the field of neurosurgery. Survey questions were presented on a 5-point Likert scale.

### Statistical analysis

All statistical analyses were performed using PRISM software (GraphPad, Boston, MA). The distance between the participants’ marking and the true craniometric point was compared between the non-AR-assisted and AR-assisted groups using two-tailed Student’s paired *t*-tests.

### Ethical considerations

All participants provided informed consent prior to participating in the study. Participants were free to withdraw from the study at any time. The study was approved by the Institutional Review Board of the university with reference number HP-00104849.

## Results

### Accuracy in identifying craniometric points

A total of 10 participants were asked to mark the location of lambda and bregma. Prior to the start of the study, participants were taught about the location of these points using a model skull. The participants then placed a marking for bregma and lambda on a cadaveric head. The cadaver’s skull was not exposed; therefore, the students had to rely on tactile feedback and measurements discussed prior to attempt. The average targeting error (standard deviation [SD]) of their markings relative to the known locations was 22.8 (10.7) mm and 11.9 (4.1) mm for lambda and bregma, respectively. Next, the participants used AR to overlay the 3D model of the cadaveric skull onto the head and placed a marking at their perceived locations of lambda and bregma. The average targeting error of their markings was 12.8 (4.9) mm and 8.6 (2.8) mm for lambda and bregma, respectively. For both craniometric points, the targeting error was significantly better in the AR-assisted group compared with the non-AR group (*p* value = 0.021 and *p* value = 0.032 for lambda and bregma, respectively, both under *p* < 0.05).

### Qualitative experience learning with AR

After completing the experiment, participants were surveyed regarding their experience with AR and its impact on their education and interest in neurosurgery. All participants felt that AR greatly improved their understanding of neuroanatomy and their ability to locate neuroanatomical structures on the cadaver (4.9/5). Overall, participants rated their experience learning using AR 4.9/5. Importantly, participants felt that the experience learning using AR increased their interest in the field of neurosurgery (4.5/5). Table 1 provides a list of qualitative data in question format as provided to the participants.

**Table 1.** AR Education Survey Questionnaire Results

Survey question	Average Likert score (SD)
Did the AR make you more interested in neurosurgery?	4.5 (53)
Did AR improve your understanding of neuroanatomy?	4.9 (0.32)
Did AR improve your understanding of the locations of key neuroanatomical landmarks?	4.7 (0.48)
Did AR help you find the landmarks on the head?	4.9 (0.32)
Did you enjoy learning with AR?	4.9 (0.32)

AR, augmented reality; SD, standard deviation.

## Discussion

### AR impact on education and interest

This study provides an early pilot feasibility study assessing the impact of AR on neurosurgical anatomy education in early learners. AR has the potential to provide a unique perspective on neurosurgery that is not available through traditional methods of instruction. By using AR, students can visualize and interact with 3D models, which can provide a better understanding of the complex anatomy and physiology of the nervous system. This may, in turn, help students to develop a deeper interest in neurosurgery, as they can see firsthand how the brain works and how it is affected by various neurological conditions. In this report, an educational exercise using AR was developed to instruct high school students about key neurosurgical anatomy and, in a larger sense, to stimulate engagement and greater interest in the field of neurosurgery. Students were instructed on the importance of craniometric points and then were asked to locate lambda and bregma on a cadaver both with and without the assistance of AR. When visualizing a 3D model of the cadaver skull overlaid onto the cadaver’s head, students were more accurate in locating bregma and lambda despite having no prior experience learning about these landmarks.

AR has emerged as a promising training tool in neurosurgery and is revolutionizing neurosurgical education by providing an immersive, interactive learning experience that enhances surgical training and decision making.^7^ AR allows residents to visualize complex neuroanatomy in three dimensions, overlaying virtual structures onto real-world models or cadavers for a more intuitive understanding of spatial relationships. This technology enables realistic, hands-on practice of intricate procedures without the risks associated with live surgery, improving precision and confidence. Additionally, AR facilitates real-time intraoperative guidance, allowing trainees to follow step-by-step overlays during procedures, which enhances learning retention and surgical accuracy. By integrating AR into neurosurgical education, residency programs are already bridging the gap between theoretical knowledge and practical skills, ultimately improving patient outcomes and surgical proficiency.

While this content can certainly be taught using textbooks and/or phantom models, the value of using AR for educational purposes is noteworthy. First, understanding 2D concepts can be challenging and thus visualization of 3D skull anatomy overlaid onto the head using AR helps students to understand these anatomical structures and concepts. Various reports have demonstrated improved knowledge scores with the use of AR for educating students on neuroanatomy.^10–12^ Second, introducing novel and exciting methods for teaching students is helpful not only to facilitate greater understanding but also to garner greater interest in the material being taught. As such, providing students with the early opportunity to get hands-on experience using AR may be an effective means to teach students as well as promote interest and appreciation for this field. Furthermore, the use of AR suggested more dynamic and engaging interactions between the neurosurgeon instructors and the students.

The survey results suggest that AR has a positive impact on students’ interest and understanding of neurosurgery. This finding is consistent with previous research that has shown the benefits of AR in enhancing engagement and motivation in various educational settings.^10^ The immersive and interactive nature of AR allows learners to actively participate in the learning process and improve communication between the instructor and students. Moreover, survey data from the study suggest that students felt that AR improved their understanding of craniometric neurosurgical landmarks. The visualization of anatomical structures in 3D using AR technology can provide students with a better understanding of the anatomical spatial relationships, which is critical for neurosurgery. Importantly, AR can also provide a platform for students to collaborate and communicate with other students and health care professionals. This can help to create a community of learners who can share their knowledge and experiences and provide feedback and support to one another.

### Study limitations

This study has some notable limitations. The sample size was small, and the study was conducted in a single institution. Therefore, the results may not generalize to other populations and settings. Additionally, the study did not test for student knowledge gains nor did it measure the long-term effects of using AR in neuroanatomy education. Therefore, this study at best serves as a pilot feasibility study to assess the ability to conduct testing, comparing AR use in neurosurgical education in new learners. Additionally, increased questioning and qualitative data capture could have been provided to assess the learner’s overall engagement and experience. Future studies exploring pretest–posttest benefits of AR for neurosurgical knowledge are warranted.

## Conclusion

This study provides a pilot feasibility study to highlight AR’s use in neurosurgical education in early, novice learners. The use of AR in education has the potential to improve interest in neurosurgery. By providing an engaging and interactive learning experience, AR can help to enhance knowledge of neuroanatomy and skills in neurosurgery as well as inspire students to possibly pursue a career in this challenging and complex field. As the technology continues to evolve, it is important to continue exploring its potential in education and health care and to identify new ways to integrate it into existing curricula. Further studies will hope to elaborate on and validate this pilot study and test its application at various levels of neurosurgical and neuroscience training.

## Abbreviations Used

3D: Three Dimensional
AR: Augmented Reality
CT: Computed Tomography
SD: Standard Deviation
